# Dietary inflammatory index and incident prostate cancer

**DOI:** 10.3389/fonc.2026.1932640

**Published:** 2026-10-07

**Authors:** Sheng Li, Yaodan Chen, Libin Zhou, Tielin Wu, Jianting Xu, Min Yin, Huimin Long, Lin Cao

**Affiliations:** 1 Department of Urology, The Affiliated Lihuili Hospital of Ningbo University, Ningbo, Zhejiang, China; 2 Departments of Urology, Ningbo Medical Center Lihuili Hospital, Ningbo, Zhejiang, China; 3 Department of Urology, Xiuzhou District People’s Hospital of Jiaxing, Jiaxing, Zhejiang, China

**Keywords:** dietary inflammatory index, prostate cancer, prospective cohort study, dietary quality, mediation analysis

## Abstract

**Background:**

The Dietary Inflammatory Index (DII) quantifies the inflammatory potential of diet. Evidence linking DII to prostate cancer remains limited and inconsistent. We evaluated the prospective association between DII and incident prostate cancer in a large population-based cohort.

**Methods:**

We included 30,972 men who were free of prostate cancer at baseline. The DII was derived from 27 dietary components assessed by the Oxford WebQ. Incident prostate cancer was identified from linked cancer-registry records (ICD-10 C61). Cox regression was the primary analysis, with age included in all adjusted models; logistic regression was secondary. Lag, additional-covariate, and age-stratified Cox models assessed sensitivity. Mediation and Mean Adequacy Ratio (MAR) analyses were exploratory.

**Results:**

During a median follow-up of 13.8 years (IQR 13.5–14.2; 406,058 person-years), 1,546 participants developed incident prostate cancer. The unadjusted inverse association (HR per 1-unit DII increase 0.951, 95% CI 0.927–0.977) was attenuated after adjustment for age and other covariates (fully adjusted HR 0.986, 95% CI 0.956–1.018; P = 0.389; Q4 vs Q1 HR 0.928, 95% CI 0.788–1.093; P for trend = 0.426). The secondary logistic analysis was likewise null (OR per 1-unit increase 0.982, 95% CI 0.950–1.015; P = 0.275). Age-adjusted lag analyses and exploratory CRP/NLR mediation analyses did not materially alter this interpretation. DII was inversely correlated with the revised 18-nutrient MAR (Pearson r = −0.694; Spearman ρ = −0.756), but neither index was associated with prostate cancer in the age-adjusted joint model.

**Conclusions:**

An apparent inverse DII–prostate cancer association in unadjusted analyses was explained largely by age differences across DII categories. After adjustment for age and other covariates, neither logistic nor time-to-event analyses supported an independent association. These findings underscore the importance of rigorous age control in prostate cancer epidemiology and should not be interpreted causally.

## 1 Introduction

Prostate cancer is the most frequently diagnosed non-cutaneous malignancy among men in high-income countries and the fifth leading cause of cancer mortality globally (1, 2). In 2022, an estimated 1.47 million new cases and approximately 397,000 deaths were attributed to prostate cancer worldwide, and incidence is projected to increase substantially in coming decades as populations age (1). The marked geographic, ethnic, and socioeconomic variation in prostate cancer incidence cannot be fully explained by genetic susceptibility alone, implicating modifiable lifestyle factors, among which diet is a plausible candidate (3, 4).

Diet influences cancer risk through multiple mechanisms, including modulation of systemic inflammation, oxidative stress, sex hormone concentrations, and the gut microbiome (5, 6). Epidemiological studies have implicated overall dietary patterns—including the Mediterranean and Western diets—in prostate cancer etiology, but findings for individual nutrients and foods have been inconsistent (7, 8). A composite index that integrates the net inflammatory potential of the total diet may better capture the diet-inflammation-cancer pathway than analyses of single foods or nutrients.

The Dietary Inflammatory Index (DII) was developed by Shivappa et al. to quantify the inflammatory potential of an individual’s diet on the basis of a comprehensive review linking 45 dietary components to circulating markers of systemic inflammation, including C-reactive protein (CRP), interleukin-6 (IL-6), and tumor necrosis factor-α (TNF-α) (9). Higher DII scores reflect a more pro-inflammatory dietary pattern. A growing body of evidence has linked higher DII scores to increased risk of several cancers, including colorectal, breast, and bladder cancer (10–12). Evidence for prostate cancer remains inconsistent: a meta-analysis of predominantly case-control studies reported a positive association (13), while individual studies have reported positive estimates (14). A 2024 meta-analysis of 16 studies reported a positive overall association between DII and prostate cancer (RR 1.52, 95% CI 1.23–1.88), but this association was driven by case-control studies (RR 1.75, 95% CI 1.34–2.28); the four prospective cohort studies showed no association (RR 1.02, 95% CI 0.96–1.08) (15), and a recent prospective cohort study in an active surveillance setting similarly reported no association between DII and grade reclassification (16). Differences in cohort characteristics, dietary assessment, follow-up, and confounder control may contribute to this heterogeneity.

An underexplored question is whether DII captures inflammatory potential alone or also reflects broader dietary quality. We therefore used the Mean Adequacy Ratio (MAR) as a supplementary measure of nutrient adequacy. This analysis was intended to characterize overlap between the indices rather than establish a causal pathway.

With over 500,000 participants recruited at 22 centers across the United Kingdom and prospectively linked to national cancer registries, it offers sufficient sample size, detailed dietary data, and robust outcome ascertainment (19). The present study aimed to: (1) examine the prospective association between DII and incident prostate cancer; (2) evaluate the robustness of this association using lag sensitivity analyses designed to reduce reverse causation bias; (3) explore whether systemic inflammation markers (CRP, NLR) mediate the association; and (4) investigate the extent to which the DII–prostate cancer association may reflect broader dietary quality as indexed by MAR.

## 2 Methods

### 2.1 Study population

This study was conducted using data from male participants enrolled in the UK Biobank, a large population-based prospective cohort study that recruited approximately 502,000 participants aged 40–69 years between 2006 and 2010 at 22 assessment centers across England, Scotland, and Wales (19). At baseline, participants completed a comprehensive assessment encompassing touchscreen questionnaires, interviewer-administered questions, physical measurements, and biological sample collection. This study received ethical approval from the North West Multi-center Research Ethics Committee (reference 11/NW/0382), and all participants provided written informed consent prior to enrolment (78619).

Eligibility was restricted to men with complete intake data for all 27 DII components who were free of prevalent prostate cancer (ICD-10 C61) at baseline. Participants with other prevalent cancers were retained because they remained at risk of incident prostate cancer; non-prostate cancers during follow-up were not censoring events. Among 501,936 participants in the linked analytic file, 70,630 had calculable DII values, including 31,595 men. We excluded 447 men with prevalent prostate cancer and 176 with missing SBP or DBP, yielding 30,972 participants. The primary reason for the reduction in sample size was the availability of sufficient dietary information required to calculate the 27-component DII from the Oxford WebQ. Because prostate cancer was the outcome of interest, the analysis was restricted to male participants. No additional 500–5,000 kcal/day exclusion was applied. The selection process is shown in **Supplementary Figure S1**.

### 2.2 Exposure assessment: dietary inflammatory index

Dietary intake was assessed using the baseline Oxford WebQ 24-hour dietary recall. Following Shivappa et al. (9), the DII was calculated from the 27 parameters available in the analytic dataset: alcohol; vitamins B12 and B6; beta-carotene; carbohydrate; cholesterol; energy; total fat; fiber; folate; iron; magnesium; monounsaturated fatty acids; niacin; n-3 fatty acids; n-6 fatty acids; protein; riboflavin; saturated fat; selenium; thiamin; trans fat; and vitamins A, C, D, and E; and zinc. Each intake was standardized to the published global mean and standard deviation, converted to a centered percentile, multiplied by its inflammatory effect score, and summed. The reduction from the original 45 parameters reflected Oxford WebQ data availability rather than outcome-driven selection. Although reduced-parameter DII versions preserve the published scoring algorithm, omission of 18 parameters may narrow exposure coverage and is acknowledged as a limitation. Component definitions are provided in **Supplementary Table S1**. Reduced-parameter DII implementations have shown construct validity against CRP in SEASONS, and a 27-parameter energy-adjusted DII has been used in UK Biobank (20, 21). These studies support feasibility but do not validate our exact component set or establish equivalence to the full 45-parameter DII. DII was analyzed continuously and in quartiles, with Q1 as the reference. Energy was retained as a DII component to preserve the scoring algorithm, whereas adjustment for total energy compared DII values at similar overall intake. This is an energy-adjusted association model, not an energy-density E-DII score. We assessed DII–energy correlation, variance inflation factors (VIFs), design-matrix rank, and the number of events per fitted coefficient (**Supplementary Table S7**).

MAR was selected because it directly quantifies nutrient adequacy using UK reference intakes and is calculable from Oxford WebQ nutrient data without importing a food-group scoring system. Eighteen nutrients were included: vitamins A, B1 (thiamin), B2 (riboflavin), B3 (niacin), B6, B12, C, and D; folate; calcium; phosphorus; iron; magnesium; zinc; selenium; copper; iodine; and potassium. These were selected because both an Oxford WebQ intake and a UK adult-male reference intake were available (17, 18). For each nutrient, the nutrient adequacy ratio was intake divided by the reference intake and truncated at 1.0; MAR was the mean of the 18 ratios multiplied by 100. Reference values and components are listed in **Supplementary Table S2**.

### 2.3 Inflammation biomarkers

CRP and NLR were considered as systemic inflammation biomarkers and were examined in the exploratory mediation analyses.

### 2.4 Outcome ascertainment

The primary outcome was incident prostate cancer, defined as a cancer-registry ICD-10 C61 diagnosis after baseline. The linked analytic file contained one cancer type and corresponding diagnosis date per participant; therefore, we used the registry diagnosis date directly and did not infer an earlier date from hospital or mortality records. Follow-up began at baseline and ended at the earliest of prostate cancer diagnosis, death, or the administrative end of cancer-registry data (11 December 2023). Other cancers were not study outcomes and were not treated as censoring events.

### 2.5 Covariates

Covariates were selected based on prior epidemiological knowledge and their potential relevance to dietary patterns and prostate cancer risk. Age at recruitment (continuous years) was included in every adjusted model because it is a major determinant of prostate cancer incidence and differed across DII quartiles. Total energy intake was included to account for overall energy intake, while ethnicity, educational attainment, household income, smoking status, and alcohol consumption were included as relevant sociodemographic and lifestyle factors. Systolic and diastolic blood pressure and diabetes status were included as additional baseline cardiometabolic characteristics. Unknown categories were retained for categorical covariates. BMI and physical activity were not included in the original covariate set and were evaluated in additional sensitivity analyses. BMI was entered continuously in kg/m² and physical activity as the baseline IPAQ activity group (low, moderate, or high). SBP, DBP, and diabetes describe baseline cardiometabolic status, but may also be downstream of dietary patterns; Model 2 was therefore retained as a comparison without these variables. This framework supports conditional association estimates, not a claim that Model 3 identifies a causal effect. Family history of prostate cancer was not included, leaving potential residual confounding.

### 2.6 Statistical analysis

Cox proportional hazards regression with time since baseline as the time scale was the primary analysis, reporting hazard ratios (HRs) and 95% confidence intervals (CIs). Logistic regression using the same covariates was secondary and reported odds ratios (ORs). Model 0 was unadjusted. Model 1 adjusted for age and total energy intake. Model 2 additionally adjusted for ethnicity, educational attainment, household income, smoking, and alcohol consumption. Model 3 additionally adjusted for SBP, DBP, and diabetes. Trend was tested by entering the quartile number as a continuous term. Cox models used Efron handling of tied event times; the same four adjustment models were used for both regression approaches, and participants were censored at death or 11 December 2023. Schoenfeld-residual tests showed no evidence of non-proportionality for continuous DII (P = 0.428), but indicated departures for age and the global model (both P < 0.001). A sensitivity Cox model stratified by single-year baseline age allowed age-specific baseline hazards while retaining all other Model 3 covariates (**Supplementary Tables S3**, **S6**).

Additional sensitivity analyses compared Model 3 with and without BMI and IPAQ activity group in the same 26,160 men with both measures and known diabetes status (1,311 events). BMI was missing in 97 men (0.31%) and IPAQ group in 4,687 (15.13%); 4,764 lacked either measure. A further 48 men with unknown diabetes status and no events were excluded from both paired models to avoid a sparse-category estimation problem; the primary cohort was unchanged. Lag sensitivity analyses excluded events occurring within 1, 2, and 3 years after baseline and re-estimated the age-adjusted fully adjusted logistic and Cox models. These prespecified intervals progressively reduce the influence of early preclinical disease without imposing a longer exclusion that would markedly reduce events. Exploratory mediation analyses used log-transformed CRP and NLR. CRP and NLR were selected as the systemic inflammatory measures available at baseline; IL-6 and TNF-α were not measured in the general cohort. Mediator and outcome models were adjusted for the Model 3 covariates, including age. Because the age-adjusted total DII association was null and exposure, mediators, and covariates were measured at baseline, these analyses were interpreted as descriptive and non-causal.

For the exploratory MAR analysis, Spearman correlation was the preferred correlation measure because it does not require bivariate normality; Pearson correlation was also reported to describe linear association. Age-adjusted logistic models evaluated MAR separately and jointly with DII.

Analyses were performed in R 4.5.2. DII construction and data management used base R and data.table; logistic models used stats::glm; Cox models and proportional-hazards diagnostics used survival::coxph and survival::cox.zph; correlations used stats::cor.test; and mediation used mediation::mediate with 500 quasi-Bayesian simulations. Two-sided P < 0.05 was considered statistically significant. The primary cohort used complete DII, SBP, and DBP data while retaining explicit unknown categories for categorical covariates. To describe dietary-data selection, included men were compared with men lacking sufficient dietary information after applying the same sex, prevalent prostate cancer, and blood-pressure eligibility criteria. Available baseline characteristics, missing counts, and standardized mean differences (SMDs) were reported descriptively, without hypothesis-test screening (**Supplementary Table S8**).

## 3 Results

### 3.1 Baseline characteristics

The analytic cohort comprised 30,972 men with a mean DII of −0.12 (SD 1.89). Follow-up ended on 11 December 2023. Median follow-up was 13.8 years (IQR 13.5–14.2), totaling 406,058 person-years. Among 1,546 incident cases, median time from baseline to diagnosis was 7.0 years (IQR 3.8–9.5); the crude incidence rate was 3.81 per 1,000 person-years.

Baseline characteristics by DII quartile are presented in **Table 1**. Mean age declined from 57.41 years in Q1 to 55.69 years in Q4, demonstrating the need for age control. Mean DII ranged from −2.60 in Q1 to 2.27 in Q4, and mean energy intake ranged from 2,762.6 to 1,714.7 kcal/day. Incident prostate cancer occurred in 5.6%, 5.1%, 4.8%, and 4.4% of Q1–Q4, respectively. Other characteristics are reported descriptively without between-group significance testing. Compared with 179,549 otherwise eligible men lacking sufficient dietary information, included men were of similar age (56.49 vs 56.69 years; SMD = −0.025) but more often held a degree (40.4% vs 32.4%; SMD = 0.167). The full comparison is provided in **Supplementary Table S8**. DII and energy were moderately correlated (Pearson r = −0.530); their VIFs were 1.449 and 1.435, respectively. The design matrix was full rank, with 1,546 events for 18 fitted coefficients (85.9 events per coefficient; **Supplementary Table S7**). **Figure 1** presents the secondary fully adjusted logistic estimates.

**Table 1:** Baseline characteristics of study participants by DII quartile (N = 30,972).

Characteristic	Overall	Q1 (Low DII)	Q2	Q3	Q4 (High DII)
N	30,972	7,743	7,743	7,743	7,743
DII, mean (SD)	−0.12 (1.89)	−2.60 (0.80)	−0.77 (0.41)	0.60 (0.40)	2.27 (0.69)
Age at recruitment (years), mean (SD)	56.49 (8.27)	57.41 (8.17)	56.70 (8.19)	56.16 (8.21)	55.69 (8.40)
Energy intake (kcal/day), mean (SD)	2,262.2 (770.4)	2,762.6 (869.9)	2,428.1 (641.1)	2,143.3 (552.8)	1,714.7 (555.7)
SBP (mmHg), mean (SD)	141.82 (18.11)	142.49 (18.09)	141.90 (17.82)	141.68 (18.31)	141.19 (18.20)
DBP (mmHg), mean (SD)	82.89 (10.62)	83.12 (10.65)	82.79 (10.56)	82.71 (10.73)	83.00 (10.55)
Ethnicity, n (%)					
White	27,640 (89.2)	6,904 (89.2)	7,019 (90.6)	6,970 (90.0)	6,747 (87.1)
Non-White	2,256 (7.3)	450 (5.8)	477 (6.2)	532 (6.9)	797 (10.3)
Unknown	1,076 (3.5)	389 (5.0)	247 (3.2)	241 (3.1)	199 (2.6)
Education, n (%)					
Degree	14,089 (45.5)	3,891 (50.2)	3,636 (47.0)	3,364 (43.4)	3,198 (41.3)
No degree	14,957 (48.3)	3,479 (44.9)	3,718 (48.0)	3,896 (50.3)	3,864 (49.9)
Unknown	1,926 (6.2)	373 (4.8)	389 (5.0)	483 (6.2)	681 (8.8)
Household income, n (%)					
High (>£100,000)	2,003 (6.5)	652 (8.4)	537 (6.9)	461 (6.0)	353 (4.6)
Middle (£31,000–£100,000)	14,609 (47.2)	3,822 (49.4)	3,797 (49.0)	3,648 (47.1)	3,342 (43.2)
Low (<£31,000)	9,688 (31.3)	2,157 (27.9)	2,401 (31.0)	2,497 (32.3)	2,633 (34.0)
Unknown	4,672 (15.1)	1,112 (14.4)	1,008 (13.0)	1,137 (14.7)	1,415 (18.3)
Smoking status, n (%)					
Never	15,204 (49.1)	3,866 (49.9)	3,847 (49.7)	3,800 (49.1)	3,691 (47.7)
Ever	14,651 (47.3)	3,519 (45.4)	3,675 (47.5)	3,700 (47.8)	3,757 (48.5)
Unknown	1,117 (3.6)	358 (4.6)	221 (2.9)	243 (3.1)	295 (3.8)
Alcohol consumption, n (%)					
Never	1,631 (5.3)	359 (4.6)	369 (4.8)	393 (5.1)	510 (6.6)
Ever	28,208 (91.1)	7,115 (91.9)	7,131 (92.1)	7,049 (91.0)	6,913 (89.3)
Unknown	1,133 (3.7)	269 (3.5)	243 (3.1)	301 (3.9)	320 (4.1)
Diabetes status, n (%)					
No	28,889 (93.3)	7,244 (93.6)	7,270 (93.9)	7,178 (92.7)	7,197 (92.9)
Yes	1,518 (4.9)	279 (3.6)	336 (4.3)	415 (5.4)	488 (6.3)
Unknown	565 (1.8)	220 (2.8)	137 (1.8)	150 (1.9)	58 (0.7)
Incident prostate cancer, n (%)	1,546 (5.0)	437 (5.6)	392 (5.1)	375 (4.8)	342 (4.4)

DII, Dietary Inflammatory Index; SBP, systolic blood pressure; DBP, diastolic blood pressure. Q1 = lowest DII quartile (most anti-inflammatory); Q4 = highest DII quartile (most pro-inflammatory). Values are mean (SD) or n (%).

**Figure 1:**
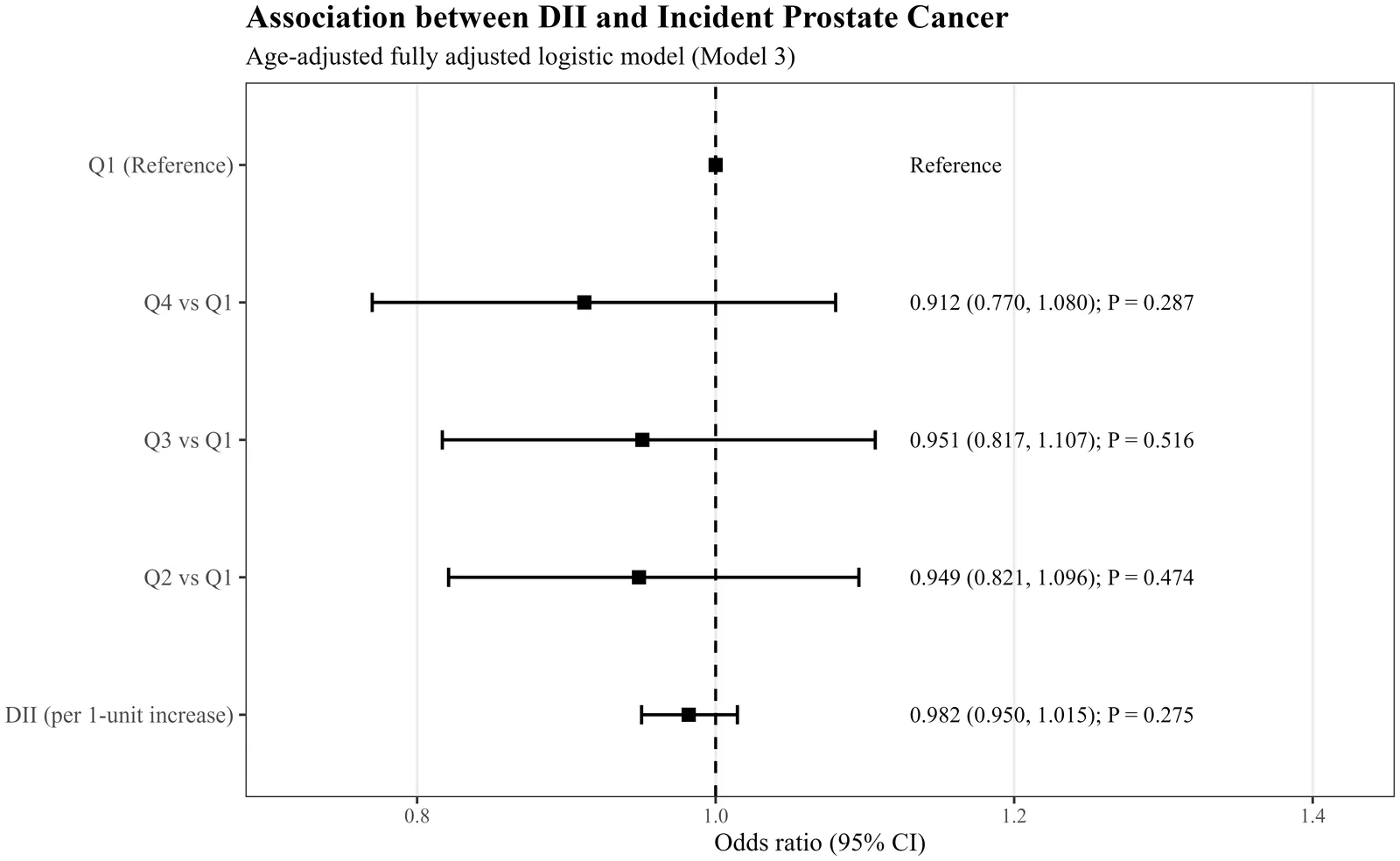
Forest plot of the secondary fully adjusted logistic association between the Dietary Inflammatory Index and incident prostate cancer. Model 3 adjusted for age, total energy intake, ethnicity, education, household income, smoking, alcohol, systolic and diastolic blood pressure, and diabetes. Q1 is the reference group for quartile comparisons. DII, Dietary Inflammatory Index; CI, confidence interval.

### 3.2 Association between DII and incident prostate cancer

In the primary Cox analysis, the unadjusted HR per 1-unit increase in DII was 0.951 (95% CI 0.927–0.977; P < 0.001), attenuating to 0.983 (0.953–1.014; P = 0.287) after age and energy adjustment. The fully adjusted HR was 0.986 (0.956–1.018; P = 0.389); for Q4 versus Q1 it was 0.928 (0.788–1.093; P for trend = 0.426; **Table 2A**). In the secondary logistic analysis (**Table 2B**), continuous DII was inversely associated with prostate cancer before adjustment (OR per 1-unit increase 0.949, 95% CI 0.924–0.975; P < 0.001). After age and energy adjustment, the association was attenuated (Model 1 OR 0.978, 95% CI 0.947–1.010; P = 0.181) and remained null in Model 3 (OR 0.982, 95% CI 0.950–1.015; P = 0.275). In Model 3, ORs were 0.949 (95% CI 0.821–1.096) for Q2, 0.951 (0.817–1.107) for Q3, and 0.912 (0.770–1.080) for Q4 versus Q1 (P for trend = 0.318). The primary Cox estimates were consistent with these secondary findings (HR per 1-unit increase 0.986, 95% CI 0.956–1.018; P = 0.389; Q4 vs Q1 HR 0.928, 95% CI 0.788–1.093; P for trend = 0.426; **Supplementary Table S3**).

**Table 2A:** Primary cox regression analysis of DII and incident prostate cancer (N = 30,972; events = 1,546).

DII	Model 0	Model 1	Model 2	Model 3
Per 1-unit increase	0.951 (0.927–0.977) P <0.001	0.983 (0.953–1.014) P = 0.287	0.987 (0.956–1.019) P = 0.417	0.986 (0.956–1.018) P = 0.389
Q1	1.000 (reference)	1.000 (reference)	1.000 (reference)	1.000 (reference)
Q2 vs Q1	0.893 (0.779–1.024) P = 0.105	0.956 (0.832–1.098) P = 0.523	0.957 (0.833–1.100) P = 0.538	0.955 (0.831–1.097) P = 0.514
Q3 vs Q1	0.856 (0.746–0.983) P = 0.028	0.966 (0.834–1.118) P = 0.640	0.971 (0.838–1.124) P = 0.689	0.966 (0.835–1.119) P = 0.648
Q4 vs Q1	0.781 (0.678–0.899) P <0.001	0.913 (0.776–1.073) P = 0.270	0.931 (0.791–1.096) P = 0.392	0.928 (0.788–1.093) P = 0.370
P for trend	<0.001	0.323	0.453	0.426

Values are HRs (95% CIs), with two-sided Wald P values. Model 0: unadjusted. Model 1: age and total energy intake. Model 2: additionally ethnicity, education, household income, smoking, and alcohol. Model 3: additionally SBP, DBP, and diabetes. Time scale: years since baseline; Efron ties. Trend uses quartile number as a continuous term. HR, hazard ratio; CI, confidence interval. The corresponding secondary logistic models are retained in **Table 2B**.

**Table 2B:** Secondary logistic analysis: association between the Dietary Inflammatory Index and incident prostate cancer across four adjustment models (N = 30,972; Cases = 1,546).

Exposure	Model 0 (Unadjusted)		Model 1 (Age + energy)		Model 2 (Sociodemo.)		Model 3 (Full)	
	OR (95% CI)	P	OR (95% CI)	P	OR (95% CI)	P	OR (95% CI)	P
DII (per 1-unit increase)	0.949 (0.924–0.975)	<0.001	0.978 (0.947–1.010)	0.181	0.983 (0.951–1.015)	0.298	0.982 (0.950–1.015)	0.275
Q1 (Reference)	1.000	—	1.000	—	1.000	—	1.000	—
Q2 vs. Q1	0.892 (0.775–1.026)	0.108	0.950 (0.822–1.097)	0.483	0.951 (0.823–1.099)	0.497	0.949 (0.821–1.096)	0.474
Q3 vs. Q1	0.851 (0.738–0.980)	0.026	0.951 (0.817–1.106)	0.512	0.956 (0.821–1.113)	0.562	0.951 (0.817–1.107)	0.516
Q4 vs. Q1	0.773 (0.668–0.893)	<0.001	0.893 (0.755–1.057)	0.188	0.915 (0.773–1.084)	0.307	0.912 (0.770–1.080)	0.287
P for trend	<0.001	—	0.220	—	0.345	—	0.318	—

Model 0: unadjusted. Model 1: adjusted for age and total energy intake. Model 2: additionally adjusted for ethnicity, education, household income, smoking, and alcohol consumption. Model 3: additionally adjusted for systolic blood pressure, diastolic blood pressure, and diabetes. OR, odds ratio; CI, confidence interval; DII, Dietary Inflammatory Index; Q, quartile.

### 3.3 Sensitivity analyses

Age-adjusted lag analyses did not support an association (**Supplementary Table S4**). After excluding events within 1, 2, and 3 years, continuous-DII ORs were 0.977 (95% CI 0.945–1.011), 0.978 (0.945–1.013), and 0.982 (0.948–1.018), respectively; corresponding Cox HRs were 0.982, 0.982, and 0.986, and all trend tests were non-significant. In the paired sample with BMI, IPAQ, and known diabetes status, the HR per 1-unit DII increase was 0.990 (95% CI 0.956–1.025; P = 0.563) before adding BMI and IPAQ and 0.992 (0.958–1.027; P = 0.633) after adding them. The baseline-age-stratified HR was 0.986 (0.955–1.018; P = 0.388; **Supplementary Table S6**).

### 3.4 Exploratory mediation analyses

Neither CRP nor NLR showed evidence of an age-adjusted indirect association (**Supplementary Table S5**). The average causal mediation effect estimates were close to zero for CRP (P = 0.128) and NLR (P = 0.824); total effects were also non-significant. Given the null total association, single-time-point measurements, and unverifiable causal assumptions, these estimates are descriptive rather than evidence for or against a biological mediation pathway.

### 3.5 Supplementary analyses with the mean adequacy ratio

DII was inversely correlated with the transparently reconstructed 18-nutrient MAR (Pearson r = −0.694; Spearman ρ = −0.756; both P < 0.001). Mean MAR values were 95.23, 92.77, 89.33, and 78.64 across Q1–Q4. In age-adjusted Model 3, MAR was not associated with prostate cancer (OR per 10-point increase 1.029, 95% CI 0.952–1.112; P = 0.467). In the joint model, both DII (OR 0.984, 95% CI 0.946–1.023; P = 0.408) and MAR (OR 1.008, 95% CI 0.920–1.105; P = 0.868) were null (**Table 3**).

**Table 3:** Supplementary analyses of the mean adequacy ratio (MAR) in relation to the dietary inflammatory index and incident prostate cancer.

Analysis	Value or OR (95% CI)	P value	N
Panel A. Correlation between DII and MAR			
Pearson correlation (DII vs. MAR)	−0.694	<0.001	30,972
Spearman correlation (DII vs. MAR)	−0.756	<0.001	30,972
Panel B. Mean MAR by DII quartile			
Q1 (lowest DII, most anti-inflammatory)	95.23	—	7,743
Q2	92.77	—	7,743
Q3	89.33	—	7,743
Q4 (highest DII, most pro-inflammatory)	78.64	—	7,743
Panel C. Association of MAR with incident prostate cancer (fully adjusted model)			
MAR per 10-point increase	1.029 (0.952–1.112)	0.467	30,972
Panel D. Joint model — DII and MAR simultaneously included			
DII (per 1-unit increase)	0.984 (0.946–1.023)	0.408	30,972
MAR (per 10-point increase)	1.008 (0.920–1.105)	0.868	30,972

MAR was calculated from 18 nutrients listed in **Supplementary Table S2**. Cancer models adjusted for age, total energy intake, ethnicity, education, household income, smoking, alcohol, systolic and diastolic blood pressure, and diabetes. MAR, Mean Adequacy Ratio; DII, Dietary Inflammatory Index; OR, odds ratio; CI, confidence interval.

## 4 Discussion

In this prospective cohort of 30,972 men, an apparent inverse DII–prostate cancer association in unadjusted analyses was substantially attenuated when age was included. The primary fully adjusted Cox model, secondary logistic model, and sensitivity analyses did not support an independent association. DII remained strongly inversely correlated with MAR, but neither index was associated with prostate cancer after age and other covariate adjustment. These results identify age as an important source of confounding in the original model and do not support an independent protective association of a more pro-inflammatory diet.

Age was unevenly distributed across DII quartiles: men in Q1 were on average 1.72 years older than those in Q4. Because prostate cancer incidence rises steeply with age, this imbalance plausibly generated the counter-intuitive crude gradient. The marked attenuation after age adjustment, together with a null Cox estimate, argues against interpreting the original inverse association as biological protection or as evidence that pro-inflammatory diets reduce prostate cancer risk.

The broader literature on DII and prostate cancer is heterogeneous, with positive, null, and inverse estimates across studies (13–16). Differences in age distributions, PSA testing, dietary instruments, available DII components, and covariate definitions may explain part of this inconsistency. The present reanalysis suggests that careful age control is particularly important when comparing DII groups in cohorts spanning midlife and older adulthood.

The strong inverse DII–MAR correlation indicates that the two indices capture overlapping dietary information, but the revised cancer models do not support independent associations for either measure. MAR should therefore be interpreted as a descriptive dietary-quality analysis rather than an explanation for prostate cancer incidence. PSA-related detection differences and residual confounding remain plausible concerns, but neither is required to explain the age-attenuated null estimate.

The exploratory CRP and NLR analyses also require restraint. Both inflammatory markers were measured once at baseline, contemporaneously with diet, and neither captures localized prostate inflammation over the long carcinogenic period. Moreover, conventional mediation quantities are not causally interpretable without strong assumptions about temporal ordering and mediator–outcome confounding; with a null age-adjusted total association, the near-zero indirect estimates are best viewed as descriptive.

Prospective evidence on circulating inflammatory markers and incident prostate cancer is less consistent than prognostic evidence in established disease. Elevated CRP and NLR have been associated more reliably with poorer outcomes after diagnosis than with the occurrence of prostate cancer. Thus, the present null exploratory estimates neither demonstrate nor exclude a role for inflammation in prostate carcinogenesis.

Excluding events occurring within the first 1–3 years did not materially change the age-adjusted null estimates. These analyses reduce the influence of early preclinical disease on reported diet but cannot eliminate reverse causation, and they may remove genuine incident cases related to baseline exposure. They should therefore be interpreted as sensitivity analyses rather than proof about causal direction.

Strengths include the large male cohort, prospective cancer-registry follow-up, a reproducible 27-component DII, explicit participant flow, age-adjusted nested models, and complementary logistic and time-to-event analyses. Reconstructing the analysis from participant identifiers also enabled correction of inconsistencies between the original descriptive table and model outputs.

Several limitations merit acknowledgement. First, dietary intake was assessed using a single 24-hour recall, which may not capture habitual long-term dietary patterns. This is particularly relevant for cancer outcomes with prolonged latency. Single recalls are subject to random within-person error, leading to non-differential misclassification of DII that typically attenuates associations towards the null. Consequently, the true DII–prostate cancer association may be stronger than observed. However, the Oxford WebQ has been validated against multiple 24-hour recalls and demonstrates acceptable ranking of habitual dietary intake in UK Biobank participants. Nevertheless, repeated dietary assessments across follow-up would have provided more accurate estimates of long-term exposure. The requirement for sufficient dietary information may have introduced selection bias if men with available DII data differed systematically from those without sufficient dietary data, which may limit the representativeness of the study population. **Supplementary Table S8** documents differences in education and other measured characteristics; this descriptive comparison cannot determine the direction or magnitude of selection bias in the DII–prostate cancer association. Second, prostate cancer ascertainment depends in part on PSA testing behavior, which is not uniformly captured in routine registry data and varies substantially across socioeconomic and demographic groups in the UK. The potential for detection bias—whereby men with anti-inflammatory dietary patterns undergo more frequent PSA testing—cannot be excluded and may partly explain the inverse association observed. Third, adjustment for baseline BMI and IPAQ activity group in sensitivity analyses did not materially change the estimates, but residual confounding by family history of prostate cancer, non-registered PSA screening, and imperfectly measured lifestyle factors remains possible. Fourth, the analytic cohort is predominantly composed of White men aged 40–69 years, limiting generalizability to younger men, other ethnic groups, and lower-income populations. Fifth, the DII was calculated from 27 of the 45 food parameters in the complete DII instrument owing to data availability constraints, potentially narrowing exposure coverage and changing individual rankings. The extent and direction of reclassification relative to the full 45-parameter score were not quantified and cannot be inferred from the reduced score alone. Sixth, the available inflammatory biomarkers (CRP, NLR) were measured at a single baseline timepoint and may not adequately reflect the long-term systemic inflammatory exposure most relevant to prostate carcinogenesis. Seventh, Excluding early incident cases reduces reverse-causation concerns but does not eliminate them, particularly over periods exceeding 3 years. This approach may also exclude cases genuinely related to baseline diet, potentially attenuating the true effect. Longer lag periods were not examined due to reduced statistical power. Therefore, the stability across lag periods should be interpreted as evidence of consistency after excluding early cases, rather than definitive proof that reverse causation has been ruled out.

In conclusion, the inverse association observed before age adjustment did not persist after appropriate control for age and other covariates or in Cox time-to-event analyses. The revised findings do not support an independent association between DII and incident prostate cancer. Replication using repeated dietary assessments, complete DII parameters, PSA-screening information, and prespecified confounder control is warranted.

## Data Availability

The original contributions presented in the study are included in the article/**Supplementary Material**. Further inquiries can be directed to the corresponding author.
