# Supplementary material for: Dietary inflammatory index and incident prostate cancer
Source: Front Oncol. 2026 Oct 7;16:1932640. doi: 10.3389/fonc.2026.1932640 (PMC13683071; doi:10.3389/fonc.2026.1932640)
Supplement: Supplementary file 1 [file DataSheet1.docx]

**Supplementary Material**

Dietary Inflammatory Index and Incident Prostate Cancer

# Supplementary Figure S1. Participant flow diagram

# Supplementary Table S1. Components used to calculate the 27-component DII

| **Component** | **Oxford WebQ variable** | **Global mean** | **Global SD** | **Inflammatory effect score** |
| --- | --- | --- | --- | --- |
| Alcohol | Alcohol | 13.98 | 3.72 | -0.278 |
| Vitamin B12 | Vitamin B12 | 5.15 | 2.7 | 0.106 |
| Vitamin B6 | Vitamin B6 | 1.47 | 0.74 | -0.365 |
| Beta Carotene | Beta-carotene | 3718 | 1720 | -0.584 |
| Carbohydrate | Carbohydrate | 272.2 | 40 | 0.097 |
| Cholesterol | Cholesterol | 279.4 | 51.2 | 0.110 |
| Energy | Energy | 2056 | 338 | 0.180 |
| Total Fat | Fat | 71.4 | 19.4 | 0.298 |
| Fiber | Englyst fibre | 18.8 | 4.9 | -0.663 |
| Folate | Folate | 273 | 70.7 | -0.190 |
| Iron | Iron | 13.35 | 3.71 | 0.032 |
| Magnesium | Magnesium | 310.1 | 139.4 | -0.484 |
| Mufa | Monounsaturated fatty acids | 27 | 6.1 | -0.009 |
| Niacin | Niacin equivalent | 25.9 | 11.77 | -0.246 |
| N-3 Fatty Acids | n-3 fatty acids | 1.06 | 1.06 | -0.436 |
| N-6 Fatty Acids | n-6 fatty acids | 10.8 | 7.5 | -0.159 |
| Protein | Protein | 79.4 | 13.9 | 0.021 |
| Riboflavin | Riboflavin | 1.7 | 0.79 | -0.068 |
| Saturated Fat | Saturated fatty acids | 28.6 | 8 | 0.373 |
| Selenium | Selenium | 67 | 25.1 | -0.191 |
| Thiamin | Thiamin | 1.7 | 0.66 | -0.098 |
| Trans Fat | Trans fatty acids | 3.15 | 3.75 | 0.229 |
| Vitamin A | Vitamin A retinol equivalents | 983.9 | 518.6 | -0.401 |
| Vitamin C | Vitamin C | 118.2 | 43.46 | -0.424 |
| Vitamin D | Vitamin D | 6.26 | 2.21 | -0.446 |
| Vitamin E | Vitamin E | 8.73 | 1.49 | -0.419 |
| Zinc | Zinc | 9.84 | 2.19 | -0.313 |

The scoring algorithm followed Shivappa et al.; only parameters available in the analytic Oxford WebQ dataset were included.

# Supplementary Table S2. Nutrients and reference intakes used for the 18-nutrient MAR

| **Nutrient** | **Unit** | **Adult-male reference intake** |
| --- | --- | --- |
| Vitamin A | µg/day | 700 |
| Thiamin (B1) | mg/day | 1 |
| Riboflavin (B2) | mg/day | 1.3 |
| Niacin (B3) | mg/day | 17 |
| Vitamin B6 | mg/day | 1.4 |
| Vitamin B12 | µg/day | 1.5 |
| Folate | µg/day | 200 |
| Vitamin C | mg/day | 40 |
| Vitamin D | µg/day | 10 |
| Calcium | mg/day | 700 |
| Phosphorus | mg/day | 550 |
| Iron | mg/day | 8.7 |
| Magnesium | mg/day | 300 |
| Zinc | mg/day | 9.5 |
| Selenium | µg/day | 75 |
| Copper | mg/day | 1.2 |
| Iodine | µg/day | 140 |
| Potassium | mg/day | 3500 |

Each nutrient adequacy ratio was intake/reference intake, capped at 1.0; MAR was the mean of the 18 ratios ×100. Reference values were based on UK Dietary Reference Values, with the vitamin D value updated to 10 µg/day in line with the 2016 SACN report.

# Supplementary Table S3. Fully adjusted primary Cox analysis and proportional-hazards diagnostics

| **Exposure** | **HR (95% CI)** | **P value** |
| --- | --- | --- |
| DII per 1-unit increase | 0.986 (0.956–1.018) | 0.389 |
| Q2 vs Q1 | 0.955 (0.831–1.097) | 0.514 |
| Q3 vs Q1 | 0.966 (0.835–1.119) | 0.648 |
| Q4 vs Q1 | 0.928 (0.788–1.093) | 0.370 |
| P for trend | — | 0.426 |

Cox models adjusted for age, total energy intake, ethnicity, education, household income, smoking, alcohol, systolic and diastolic blood pressure, and diabetes. Administrative end date: 11 December 2023. Schoenfeld-residual tests: continuous DII P=0.428, age P=1.23e-12 and global P=2.23e-7; for the quartile model, DII P=0.569, age P=1.23e-12 and global P=5.59e-7. These tests do not support a blanket claim that all proportional-hazards assumptions were satisfied. An age-stratified sensitivity analysis is reported in Supplementary Table S6. Staged primary Cox models are presented in Table 2A.

# Supplementary Table S4. Age-adjusted lag sensitivity analyses

| **Excluded interval** | **N** | **Events** | **Logistic OR per unit** | **P** | **Q4 vs Q1 OR** | **P** | **Cox HR per unit** | **P** | **Logistic P trend** |
| --- | --- | --- | --- | --- | --- | --- | --- | --- | --- |
| 1 year | 30,826 | 1,463 | 0.977 (0.945–1.011) | 0.180 | 0.900 (0.756–1.070) | 0.232 | 0.982 (0.950–1.014) | 0.268 | 0.237 |
| 2 year | 30,621 | 1,362 | 0.978 (0.945–1.013) | 0.210 | 0.904 (0.756–1.082) | 0.271 | 0.982 (0.950–1.016) | 0.292 | 0.258 |
| 3 year | 30,432 | 1,259 | 0.982 (0.948–1.018) | 0.333 | 0.915 (0.760–1.102) | 0.349 | 0.986 (0.952–1.021) | 0.426 | 0.393 |

All models used the fully adjusted covariate set including age.

# Supplementary Table S5. Exploratory age-adjusted mediation analyses

| **Mediator** | **N** | **ACME (95% CI)** | **P** | **ADE (95% CI)** | **P** | **Total effect (95% CI)** | **P** |
| --- | --- | --- | --- | --- | --- | --- | --- |
| CRP | 29,154 | -0.000102 (-0.000240, 0.000020) | 0.128 | -0.000474 (-0.001922, 0.000995) | 0.552 | -0.000577 (-0.002090, 0.000884) | 0.452 |
| NLR | 29,777 | 0.000002 (-0.000017, 0.000021) | 0.824 | -0.000594 (-0.002043, 0.000845) | 0.440 | -0.000592 (-0.002053, 0.000848) | 0.440 |

Mediator and outcome models included age and all Model 3 covariates. Estimates used 500 quasi-Bayesian simulations and are descriptive because the age-adjusted total association was null and causal mediation assumptions were not verified.

# Supplementary Methods: software and reproducibility

Analyses were run in R 4.5.2. Data management and score construction used data.table; logistic regression used stats::glm; correlations used stats::cor.test; Cox regression and proportional-hazards diagnostics used survival::coxph and survival::cox.zph; and exploratory mediation used mediation::mediate with 500 quasi-Bayesian simulations. The accompanying source files include the score definitions, participant-flow counts, model estimates, figure source data, and the R scripts used during revision.

**Supplementary Table S6. Additional covariate and age-stratified Cox sensitivity analyses**

| **Analysis / exposure** | **N / events** | **HR (95% CI)** | **P** |
| --- | --- | --- | --- |
| Paired Model 3 / per 1-unit DII | 26,160 / 1,311 | 0.990 (0.956–1.025) | 0.563 |
| Paired Model 3 / Q2 vs Q1 | 26,160 / 1,311 | 0.993 (0.855–1.153) | 0.922 |
| Paired Model 3 / Q3 vs Q1 | 26,160 / 1,311 | 1.003 (0.856–1.176) | 0.970 |
| Paired Model 3 / Q4 vs Q1 | 26,160 / 1,311 | 0.948 (0.792–1.133) | 0.556 |
| Paired Model 3 + BMI/IPAQ / per 1-unit DII | 26,160 / 1,311 | 0.992 (0.958–1.027) | 0.633 |
| Paired Model 3 + BMI/IPAQ / Q2 vs Q1 | 26,160 / 1,311 | 0.994 (0.855–1.154) | 0.935 |
| Paired Model 3 + BMI/IPAQ / Q3 vs Q1 | 26,160 / 1,311 | 1.006 (0.858–1.180) | 0.938 |
| Paired Model 3 + BMI/IPAQ / Q4 vs Q1 | 26,160 / 1,311 | 0.955 (0.798–1.143) | 0.617 |
| Age-stratified Model 3 / per 1-unit DII | 30,972 / 1,546 | 0.986 (0.955–1.018) | 0.388 |
| Age-stratified Model 3 / Q2 vs Q1 | 30,972 / 1,546 | 0.948 (0.825–1.090) | 0.455 |
| Age-stratified Model 3 / Q3 vs Q1 | 30,972 / 1,546 | 0.962 (0.831–1.114) | 0.605 |
| Age-stratified Model 3 / Q4 vs Q1 | 30,972 / 1,546 | 0.928 (0.788–1.093) | 0.371 |

Paired analyses required complete BMI and IPAQ activity group and known diabetes status: 4,764 men lacked BMI or IPAQ (BMI: 97/30,972, 0.31%; IPAQ: 4,687/30,972, 15.13%), and 48 further men had unknown diabetes status and no events. Excluding the same 48 men from both paired models avoided a sparse-category estimation problem. Other categorical Unknown groups were retained. BMI was continuous (kg/m²), IPAQ low/moderate/high, with low as reference. Age-stratified Cox regression used separate baseline hazards for each single-year baseline-age stratum instead of a linear age coefficient, retaining all other Model 3 covariates and all 30,972 men. Original-cohort DII quartile cutpoints were retained. All analyses are sensitivity analyses; P values are nominal.

**Supplementary Table S7. Energy adjustment and collinearity diagnostics**

| **Diagnostic** | **Value** |
| --- | --- |
| Pearson r: DII and energy | −0.530 |
| Spearman rho: DII and energy | −0.533 |
| DII VIF | 1.449 |
| Energy VIF | 1.435 |
| Largest treatment-indicator VIF | 4.387 |
| Design matrix rank / columns (including intercept) | 19 / 19 |
| Events / fitted Cox coefficients | 1,546 / 18 |
| Events per coefficient | 85.9 |
| age_at_recruitment VIF | 1.298 |
| ethnicity3Unknown VIF | 1.107 |
| ethnicity3White VIF | 1.184 |
| education3No degree VIF | 1.238 |
| education3Unknown VIF | 1.310 |
| income4Low VIF | 4.387 |
| income4Middle VIF | 4.189 |
| income4Unknown VIF | 2.197 |
| smoking3Never VIF | 1.052 |
| smoking3Unknown VIF | 1.091 |
| alcohol3Never VIF | 1.060 |
| alcohol3Unknown VIF | 1.103 |
| sbp VIF | 2.096 |
| dbp VIF | 1.948 |
| diabetes3Unknown VIF | 1.052 |
| diabetes3Yes VIF | 1.033 |

VIFs were calculated from the Model 3 design matrix using one column per treatment-coded factor indicator and continuous covariate. Thus factor-indicator VIFs, not generalized VIFs for entire multi-level factors, are shown. The intercept is included only in the rank calculation; the Cox model contains 18 fitted regression coefficients. These diagnostics assess linear dependence and model size, not absence of residual confounding, selection bias, or overfitting.

**Supplementary Table S8. Included and otherwise eligible dietary-excluded men**

| **Characteristic** | **Included (N=30,972)** | **Dietary-excluded (N=179,549)** | **SMD** |
| --- | --- | --- | --- |
| Age, years | 56.49 (8.27) | 56.69 (8.18) | -0.025 |
| Energy, kcal/day | 2262.15 (770.35) | Not available | — |
| Energy, kcal/day: missing | 0 | 179,549 | — |
| SBP, mmHg | 141.82 (18.11) | 142.81 (18.59) | -0.054 |
| DBP, mmHg | 83.53 (10.46) | 84.10 (10.59) | -0.054 |
| BMI, kg/m² | 27.64 (4.18) | 27.86 (4.26) | -0.054 |
| BMI, kg/m²: missing | 97 | 1,002 | — |
| Ethnicity: Non-White | 1858 (6.0%) | 9886 (5.5%) | 0.021 |
| Ethnicity: Unknown | 169 (0.5%) | 1070 (0.6%) | -0.007 |
| Ethnicity: White | 28945 (93.5%) | 168593 (93.9%) | -0.018 |
| Education: Degree | 12525 (40.4%) | 58206 (32.4%) | 0.167 |
| Education: No degree | 14986 (48.4%) | 85775 (47.8%) | 0.012 |
| Education: Unknown | 3461 (11.2%) | 35568 (19.8%) | -0.240 |
| Income: High | 2173 (7.0%) | 9228 (5.1%) | 0.079 |
| Income: Low | 11105 (35.9%) | 72651 (40.5%) | -0.095 |
| Income: Middle | 15060 (48.6%) | 76345 (42.5%) | 0.123 |
| Income: Unknown | 2634 (8.5%) | 21325 (11.9%) | -0.112 |
| Smoking: Ever | 15055 (48.6%) | 91889 (51.2%) | -0.051 |
| Smoking: Never | 15822 (51.1%) | 86622 (48.2%) | 0.057 |
| Smoking: Unknown | 95 (0.3%) | 1038 (0.6%) | -0.041 |
| Alcohol: Ever | 30214 (97.6%) | 173883 (96.8%) | 0.043 |
| Alcohol: Never | 726 (2.3%) | 5120 (2.9%) | -0.032 |
| Alcohol: Unknown | 32 (0.1%) | 546 (0.3%) | -0.045 |
| Diabetes: No | 28854 (93.2%) | 165800 (92.3%) | 0.032 |
| Diabetes: Unknown | 90 (0.3%) | 1024 (0.6%) | -0.043 |
| Diabetes: Yes | 2028 (6.5%) | 12725 (7.1%) | -0.021 |
| IPAQ activity group: high | 11242 (36.3%) | 61522 (34.3%) | 0.043 |
| IPAQ activity group: low | 4681 (15.1%) | 27758 (15.5%) | -0.010 |
| IPAQ activity group: moderate | 10362 (33.5%) | 54899 (30.6%) | 0.062 |
| IPAQ activity group: Unknown | 4687 (15.1%) | 35370 (19.7%) | -0.121 |

Both groups are men without prevalent C61 and with complete SBP/DBP in the same linked file. The included group has all 27 DII components; the comparison group lacks sufficient dietary information. Means (SDs) use available observations; categorical cells are n (% of all men in that group), with Unknown shown explicitly. Unknown includes missing/indeterminate responses under the original coding, including education responses coded Unknown; it is not necessarily item non-response. Energy was unavailable for all dietary-excluded men. SMD = (included mean or proportion − excluded mean or proportion) divided by the pooled SD; categorical SMDs are level-specific. Comparison variables were not selected by statistical significance. These differences cannot identify the direction or magnitude of selection bias in the estimated association.
